# *Eikenella corrodens* Sepsis with Cerebrospinal Fluid Pleocytosis in a Very Low Birth Weight Neonate

**DOI:** 10.1155/2015/686812

**Published:** 2015-11-09

**Authors:** Christopher Sawyer, Dimitrios Angelis, Robert Bennett

**Affiliations:** Division of Neonatology, Department of Pediatrics, Texas Tech University Health Sciences Center, Odessa, TX 79763, USA

## Abstract

We report a case of* Eikenella corrodens* sepsis associated with CSF pleocytosis in a very low birth weight neonate. A 1000-gram male neonate was born at 27-week gestation due to preterm labor. The patient presented with signs and symptoms of sepsis and was treated for suspected meningitis with intravenous ampicillin and gentamicin for 7 days, with cefotaxime added for three weeks. He had a normal brain MRI at discharge and normal development at 6 months of life. To our knowledge, this is the first case of* E. corrodens* sepsis and associated meningitis in a very low birth weight neonate.

## 1. Introduction

*Eikenella corrodens* is a gram negative rod which is a normal inhabitant of human gastrointestinal tract, including the oral cavity.* E. corrodens* is primarily an opportunistic pathogen capable of causing a wide range of diseases. Presented here is a case of neonatal sepsis in a 27-week gestational age male newborn associated with cerebrospinal fluid pleocytosis.

## 2. Case Presentation

A 1000-gram male neonate was born at 27-week gestation to a previously healthy mother. Routine prenatal labs were unremarkable except group B streptococcus (GBS) status, which was unknown. The mother presented in preterm labor and a male infant was delivered precipitously, at a referring hospital. Rupture of membranes occurred spontaneously one hour prior to delivery, with the amniotic fluid reported as clear. One hour prior to delivery the mother received prenatal steroids as well as one dose of ampicillin intravenously. From the maternal history, there was no reported fever, abdominal pain, weight loss, or other constitutional symptoms.

Following delivery, the baby developed respiratory distress and apnea. Initial resuscitation efforts included positive pressure ventilation and intubation at approximately 3 minutes of life. Apgar scores were 4 and 7, at 1 and 5 minutes, respectively. An initial chest radiograph was suggestive of respiratory distress syndrome; 1 dose of surfactant was subsequently given and infant was placed on mechanical ventilation. Prior to transport, a blood culture was obtained and ampicillin and gentamicin were initiated for suspected neonatal sepsis. He remained on mechanical ventilation for 1 day and was subsequently extubated to nasal continuous positive airway pressure (nCPAP). Initial complete blood count, at about six hours of life, was significant for a low total white blood cell count with left shift. Specifically, WBCs were 4800/*μ*L with a differential count: segmented PMN 19%, bands 30%, metamyelocytes 4%, lymphocytes 30%, and monocytes 17%. The I : T ratio was 0.64 (normal < 0.2). On admission, the platelets were 240,000/*μ*L and the hemoglobin was 13 grams/dL. Initial CRP was 1.3 mg/dL (normal < 1 mg/dL) and the peak value, which occurred on day of life 2 (DOL 2), was 2.3 mg/dL.

On DOL 3 the initial blood culture obtained at the referring hospital grew* E. corrodens*. This finding triggered a complete evaluation for sepsis including a repeat blood culture as well as a lumbar puncture and urine culture. The cerebrospinal fluid (CSF) findings revealed pleocytosis and specifically CSF red blood cells (RBCs) 24800/*μ*L and white blood cells (WBCs) 990/*μ*L (58% segmented PMN, 2% bands, 35% lymphocytes, and 5% monocytes). However, gram stain and culture remained negative. The total protein and the glucose in CSF were within normal limits for the degree of prematurity. A blood culture was repeated for three consecutive days, 24 hours apart, all of which remained negative. The urine analysis was negative for white blood cells and the culture remained negative. Due to the initial blood culture, and after consultation with an infectious disease specialist, cefotaxime was added to the initial antibiotic regimen and the overall antibiotic regimen was readjusted as follows: ampicillin (100 mg/kg/dose every 12 hours), gentamicin (4.5 mg/kg/dose every 36 hours), and cefotaxime (50 mg/kg/dose every 12 hours). Ampicillin and gentamicin were discontinued after 7 days of treatment (ensuring that the subsequently obtained blood cultures remained negative) while he received a 3-week course of cefotaxime. Due to low birth weight, prematurity, use of prolonged course of antibiotics, and the presence of a central catheter, it was felt that the infant was at high risk for developing invasive fungemia and hence fluconazole prophylaxis was initiated at a dose of 3 mg/kg/dose every 72 hours intravenously. Fluconazole was discontinued upon completion of the antibiotic treatment and removal of the peripherally inserted central catheter. An echocardiogram was performed and ruled out endocarditis. The remaining hospital course was unremarkable. He was discharged home on DOL 60 (36-week corrected age), neurologically appropriate, and weighing 2300 grams. A brain MRI was obtained prior to discharge and was normal. At six-month follow-up, the patient was developmentally appropriate for age.

## 3. Discussion

Neonatal sepsis is a systemic infection that occurs early in an infant's life. Early onset sepsis (EOS) develops within 72 hours of life and is typically attributed to pathogens acquired perinatally. GBS and* E. coli* account for more than 70% of cases of EOS. On occasion, rare opportunistic pathogens are the cause of EOS. We describe here a case of EOS caused by* Eikenella corrodens* in an extremely preterm male neonate.* E. corrodens* is found in the oral cavity as well as the gastrointestinal tract of both humans and animals. It is a small, nonmotile, gram negative rod which occasionally can appear as coccobacillus.* E. corrodens* is known as the corroding bacteria, named for the phenomenon where colonies of the bacteria form characteristic pits on agar plates.* E. corrodens* is a facultative anaerobe and is generally only able to grow on blood or chocolate agar with 3–10% CO_2_ [1]. The paucity of cases of neonatal infections involving* E. corrodens* could be partially explained by the difficulty in isolating and culturing the bacteria. Molecular techniques, such as DNA hybridization, have been also used for the identification of several pathogens of the oral cavity, including* E. corrodens* [2].

*E. corrodens* has been implicated in a variety of infections, including head and neck infections in those with local cancer, osteomyelitis (particularly secondary to bite wounds), endocarditis (a member of the HACEK group, predominantly found in intravenous drug users who lick their needles), meningitis, intra-abdominal infections, suppurative thyroiditis, and neonatal conjunctivitis [3–5]. In all these cases, antibiotic treatment is deemed necessary.* E. corrodens* is commonly susceptible to penicillin, ampicillin, piperacillin, and both second- and third-generation cephalosporins.* E. corrodens* is commonly resistant to antistaphylococcal penicillins such as methicillin and nafcillin [6]. Additionally, beta-lactamase expression has been reported, raising the potential issue of resistance to penicillins. As a result, third-generation cephalosporins, such as cefotaxime, are good first-line antibiotics [7].

*E. corrodens* is a rare cause of neonatal sepsis. A literature review shows seven documented cases of neonatal sepsis since 1985 [8–13]. All cases described occurred in premature infants, where the presenting symptom was predominantly preterm labor and associated neonatal sepsis. The median gestational age of the reviewed cases was 30 weeks. In all the reviewed cases the outcome was favorable except in one case of a 24-week gestational age neonate that did not survive beyond the immediate neonatal period [13]. One possible risk factor for the development of maternofetal infection with* E. corrodens* is the presence of advanced periodontal disease in the mother. Periodontitis has been shown to cause bacteremias by oral bacteria, including some of the HACEK (*Haemophilus* species,* Actinobacillus, Cardiobacterium hominis, Eikenella corrodens,* and* Kingella*) group, either following surgical procedures or spontaneously, and is therefore associated with a variety of diseases that have significant impact in early childhood, as well as in adult life [14, 15]. Several studies have shown that periodontitis can have a significant impact on the placental-fetal unit, exacerbate a fetal inflammatory response, and lead to preterm delivery [16]. In our case, although we were not able to elicit a history of periodontitis, we could speculate that this could be a contributing factor given the presence of* E. corrodens* and preterm labor. The risk of maternofetal infection can also be associated with close contact with animals [8, 9].

Our patient exhibited symptoms and signs of sepsis and he was evaluated for associated meningitis although he did not present with typical neurologic symptoms such as seizures and severe apnea. His initial hospital course was complicated with respiratory depression requiring mechanical ventilation on day of life 0, with improvement afterwards. The organism was not isolated from the spinal fluid despite the significant CSF pleocytosis. Meningitis in this case was presumed based on the CSF analysis but not proven by CSF culture. A possible explanation for this result includes receiving multiple doses of antibiotics prior to CSF analysis and culture. Additionally, although* E. corrodens* has been shown to cause meningitis and brain abscesses in adults [3], as well as chronic meningitis in immunocompromised hosts [17], it has never been documented in neonates, to our knowledge. The CSF analysis showed an increased red blood cell count, a common finding when interpreting neonatal CSF samples [18]. It has been shown that in neonates almost 50% of CSF examinations return results with high red blood cell counts [19], interfering with the interpretation of these studies [20]. The patient had been diagnosed with a low grade intraventricular hemorrhage at birth, which could also have contributed to the presence of RBCs in the analysis that we performed, although sequelae of this finding were not confirmed on MRI prior to discharge.

Standard treatment of neonatal infections, specifically with ampicillin, provides coverage of* E. corrodens*. Furthermore, in the event of preterm labor and suspicion of chorioamnionitis, routine treatment with ampicillin prior to delivery may have eradicated the penicillin sensitive* E. corrodens* strains and have contributed to the negative blood and CSF cultures during evaluation of the neonate. In similar cases, different techniques, such as polymerase chain reaction or mass spectrometry, may be more helpful in identifying neonates with* E. corrodens* culture negative sepsis [21, 22].

## 4. Conclusion

*E. corrodens* is an opportunistic pathogen which can cause sepsis in both adults and children. It is usually associated with characteristic risk factors. Symptoms of neonatal sepsis caused by* E. corrodens* are nonspecific, making diagnosis on clinical grounds challenging. Culture proven cases attributed to* E. corrodens* have been rarely reported. Interestingly, there is a paucity of cases of CNS involvement of* E. corrodens* in neonatal literature. It is reassuring that the majority of strains of* E. corrodens* are susceptible to ampicillin, part of standard treatment in early onset sepsis. Typically, the pathogen is susceptible to cephalosporins also, which are frequently used to treat the infection and yield favorable results. Our patient presented with bacteremia in addition to presumed meningitis and was successfully treated with a three-week antibiotic regimen, including initially ampicillin and gentamicin and later a third-generation cephalosporin. Further research into what risks and events predispose to* E. corrodens* maternofetal infections could lead to both prevention and identification of this specific pathogen prior to culture results.

## References

[1] Decker M. D. (1986). Eikenella corrodens. *Infection Control*.

[2] Buduneli N., Baylas H., Buduneli E., Türkoğlu O., Köse T., Dahlen G. (2005). Periodontal infections and pre-term low birth weight: a case-control study. *Journal of Clinical Periodontology*.

[3] Emmerson A. M., Mills F. (1978). Recurrent meningitis and brain abscess caused by *Eikenella corrodens*. *Postgraduate Medical Journal*.

[4] Chhabra M. S., Motley W. W., Mortensen J. E. (2008). Eikenella corrodens as a causative agent for neonatal conjunctivitis. *Journal of AAPOS*.

[5] Yoshino Y., Inamo Y., Fuchigami T. (2010). A pediatric patient with acute suppurative thyroiditis caused by *Eikenella corrodens*. *Journal of Infection and Chemotherapy*.

[6] Sofianou D., Kolokotronis A. (1990). Susceptibility of Eikenella corrodens to antimicrobial agents. *Journal of Chemotherapy*.

[7] Goldstein E. J., Cherubin C. E., Corrado M. L., Sierra M. F. (1982). Comparative susceptibility of *Yersinia enterocolitica*, *Eikenella corrodens*, and penicillin-resistant and penicillin-susceptible *Streptococcus pneumoniae* to *β*-lactam and alternative antimicrobial agents. *Reviews of Infectious Diseases*.

[8] Hu B. L., Crewalk J. M., Ascher D. P. (2012). Congenital sepsis caused by *Eikenella corrodens*. *Open Journal of Pediatrics*.

[9] Jadhav A. R., Belfort M. A., Dildy G. A. (2009). *Eikenella corrodens* chorioamnionitis: modes of infection?. *American Journal of Obstetrics & Gynecology*.

[10] Garnier F., Masson G., Bedu A. (2009). Maternofetal infections due to *Eikenella corrodens*. *Journal of Medical Microbiology*.

[11] Andrés M. T., Martín M. C., Fierro J. F., Méndez F. J. (2002). Chorioamnionitis and neonatal septicaemia caused by Eikenella corrodens. *Journal of Infection*.

[12] Jeppson K. G., Reimer L. G. (1991). Eikenella corrodens chorioamnionitis. *Obstetrics and Gynecology*.

[13] Sporken J. M. J., Muytjens H. L., Vemer H. M. (1985). Intra-uterine infection due to eikenella corrodens. *Acta Obstetricia et Gynecologica Scandinavica*.

[14] Parahitiyawa N. B., Jin L. J., Leung W. K., Yam W. C., Samaranayake L. P. (2009). Microbiology of odontogenic bacteremia: beyond endocarditis. *Clinical Microbiology Reviews*.

[15] Pizzo G., Guiglia R., Russo L. L., Campisi G. (2010). Dentistry and internal medicine: from the focal infection theory to the periodontal medicine concept. *European Journal of Internal Medicine*.

[16] Bobetsis Y. A., Barros S. P., Offenbacher S. (2006). Exploring the relationship between periodontal disease and pregnancy complications. *Journal of the American Dental Association*.

[17] Dong X.-Y., Gong L. (2013). Chronic meningitis caused by *Eikenella corrodens*. *Kaohsiung Journal of Medical Sciences*.

[18] Nigrovic L. E., Shah S. S., Neuman M. I. (2011). Correction of cerebrospinal fluid protein for the presence of red blood cells in children with a traumatic lumbar puncture. *Journal of Pediatrics*.

[19] Greenberg R. G., Smith P. B., Cotten C. M., Moody M. A., Clark R. H., Benjamin D. K. (2008). Traumatic lumbar punctures in neonates: test performance of the cerebrospinal fluid white blood cell count. *Pediatric Infectious Disease Journal*.

[20] Pacatte K. (2008). Analysis of cerebrospinal fluid in the neonate. *Neonatal Network*.

[21] Laforgia N., Coppola B., Carbone R., Grassi A., Mautone A., Iolascon A. (1997). Rapid detection of neonatal sepsis using polymerase chain reaction. *Acta Paediatrica*.

[22] Clerc O., Prod'hom G., Senn L. (2014). Matrix-assisted laser desorption ionization time-of-flight mass spectrometry and PCR-based rapid diagnosis of Staphylococcus aureus bacteraemia. *Clinical Microbiology and Infection*.

